# Integrative analysis identifies key genes related to metastasis and a robust gene-based prognostic signature in uveal melanoma

**DOI:** 10.1186/s12920-022-01211-1

**Published:** 2022-03-17

**Authors:** Shizhen Lei, Yi Zhang

**Affiliations:** 1 Department of Ophthalmology, The Fourth Affiliated Hospital of China Medical University, Shenyang, 110032 China; 2 Department of Gerontology and Geriatrics, Shengjing Hospital of China Medical University, 36 Sanhao Road, Shenyang, 110004 China

**Keywords:** Uveal melanoma, TCGA, GEO, Prognosis, Weighted gene co-expression network analysis, Tumor microenvironment

## Abstract

**Purpose:**

Uveal melanoma (UM) is an aggressive intraocular malignancy, leading to systemic metastasis in half of the patients. However, the mechanism of the high metastatic rate remains unclear. This study aimed to identify key genes related to metastasis and construct a gene-based signature for better prognosis prediction of UM patients.

**Methods:**

Weighted gene co-expression network analysis (WGCNA) was used to identify the co-expression of genes primarily associated with metastasis of UM. Univariate, Lasso-penalized and multivariate Cox regression analyses were performed to establish a prognostic signature for UM patients.

**Results:**

The tan and greenyellow modules were significantly associated with the metastasis of UM patients. Significant genes related to the overall survival (OS) in these two modules were then identified. Additionally, an OS-predicting signature was established. The UM patients were divided into a low- or high-risk group. The Kaplan–Meier curve indicated that high-risk patients had poorer OS than low-risk patients. The receiver operating curve (ROC) was used to validate the stability and accuracy of the final five-gene signature. Based on the signature and clinical traits of UM patients, a nomogram was established to serve in clinical practice.

**Conclusions:**

We identified key genes involved in the metastasis of UM. A robust five-gene‐based prognostic signature was constructed and validated. In addition, the gene signature-based nomogram was created that can optimize the prognosis prediction and identify possible factors causing the poor prognosis of high-risk UM patients.

**Supplementary Information:**

The online version contains supplementary material available at 10.1186/s12920-022-01211-1.

## Introduction

Uveal melanoma (UM) is the most common primary intraocular malignant tumor in adults [1]. Ocular treatment for UM includes radiotherapy, phototherapy, local resection, and enucleation. Notably, almost half of UM patients will develop systemic metastasis despite successful local treatment [2]. In the United States alone, the morbidity of UM patients is nearly 5.1 per million [3]. The liver is the common site for systemic metastasis in UM, contributing to overall mortality within 1 year after confirming as metastases [1]. Moreover, effective therapies to prevent the development of metastases are not yet available, besides the lack of powerful tools for predicting the prognosis of UM patients. Therefore, new biomarkers for predicting the prognosis are urgently warranted.

Weighted gene co-expression network analysis (WGCNA) is an algorithm that analyzes the expression patterns of multiple genes and the association between the genes and clinical traits [4, 5]. In our study, WGCNA was explored to identify the co-expression modules significantly related to the survival time and metastasis of UM patients. Additionally, we identified the hub genes in the modules using the String database and Cytoscape software. Then we constructed a 5‐gene‐based signature for predicting the overall survival (OS) of UM patients using Cox regression analyses.

The tumor microenvironment (TME) plays a critical role in the progression of tumors. The TME characteristics are associated with the prognosis and drug sensitivity in cancers patients [6, 7]. Infiltrating immune cells in the TME of tumors also influence the phenotype of tumor cells, thus deciding the fate of tumor progression [8]. Previous studies have identified that infiltrating T cells in TME was a prognosis-predicting factor for UM [9, 10]. ESTIMATE (Estimation of STromal and Immune cells in MAlignant Tumor tissues using Expression data) [11] and CIBERSORT (Cell-type Identification By Estimating Relative Subsets Of RNA Transcripts) [12] are algorithms for evaluating and quantifying the infiltration of immune cells in tumor tissues. Here, we used these two algorithms to identify the differences in immune infiltration status between the low- and high-risk UM samples. The risk groups were classified by our 5‐gene‐based risk score formula. Furthermore, the correlation between the five genes in our signature and survival-related immune cells was investigated.

## Materials and methods

### Dataset collection

We extracted mRNA expression profiles and the corresponding clinical characteristics of UM samples from Gene Expression Omnibus (GEO) database (https://www.ncbi.nlm.nih.gov/geo/) including GSE22138 and GSE44295. After eliminating samples without necessary data, we finally obtained 63 UM samples from GSE22138 and 57 from GSE44295. In addition, we downloaded the mRNA expression profiles along with the clinical traits of 80 UM samples from The Cancer Genome Atlas (TCGA) database (https://portal.gdc.cancer.gov/). We used GSE22138 dataset as the training set, and the GSE44295 and TCGA-UM datasets as the testing sets.

### Standardization of the expression data

The expression data of genes in the training set and testing sets was standardized through the limma [13] package in R software. Furthermore, we used the sva [14] and limma R packages to remove the batch effect between the training set and the testing sets.

### Construction of the co-expression modules

To identify more significant genes, only the top quarter of the most variably expressed genes among 63 UM samples in the training cohort are incorporated into subsequent construction of the co-expression modules. The weighted gene co-expression network analysis (WGCNA) method based on R software package “WGCNA” was used to construct the co-expression modules [5]. The developer of the WGCNA algorithm used an adjacency matrix method to construct correlation network of genes and further correlate the gene co-expression modules with clinical traits. We chose 4 as the soft-thresholding power when 0.9 was used as the correlation coefficient threshold. The minimum number of genes in co-expression modules we chose was 20. We set 0.25 as the cut height threshold to merge possible similar modules. As for the selection of the modules to be taken as metastasis-related module, we set 0.3 as the coefficient threshold for module-trait relationship (> 0.3 means positively related to metastasis and < − 0.3 means negatively associated with metastasis). The gene significance obtained in WGCNA means the correlation between a gene and a clinical trait and high gene significance means this gene was highly correlated with the clinical trait.

### Functional enrichment analysis

Gene Ontology (GO) and Kyoto Encyclopedia of Genes and Genomes (KEGG) [15–17] enrichment analyses were performed by using the Metascape database (https://metascape.org/) to obtain further insights into the functions of genes in co-expression modules [18]. The Metascape database is a useful online tool that could return the functional annotation results of genes after directly inputting the gene list into the webpage of Metascape database. The cutoff for significance was set as *P* value < 0.05.

### Identification of hub genes in the modules

The String database is an online tool for investigating the interactions between genes and can return an interaction network of genes by directly inputting a gene list into the webpage. We used the String database (https://string-db.org) [19] to obtain an interaction network of the genes in the modules of interest. After that, we could input the file of the network obtained from the String database into the Cytoscape software [20], which is widely used for the investigation of genes and gene–gene interactions in cancer research. Moreover, there is a useful and widely used tool (cytohubba) inside the Cytoscape software for identifying the key nodes (genes) in a gene interaction network [21, 22] and we applied this tool to identify the top 50 hub genes in the two modules of interest. There are several methods inside the cytohubba tool and we chose the MCC method to identify hub genes, which could be achieved by simple selection on the page of the Cytoscape software.

### Construction and verification of the prognostic signature

We evaluated the prognostic value of the hub genes by the univariate Cox regression analysis through the R package “survival” [23]. The survival-related genes (*P* < 0.05) were enrolled into the subsequent Lasso-penalized and multivariate Cox regression analyses. The Lasso-penalized Cox regression analysis was performed in the R software by using the “glmnet” and the “survival” R package. After preparing the file containing the survival time and the expression profiles of genes, the Lasso-penalized Cox regression analysis could calculate the value of the partial likelihood deviance and the corresponding lambda value during the cross validation. The smaller the value of the partial likelihood deviance is, the better the performance of the model will be. Therefore, we chose the lambda value with lowest corresponding deviance and this algorithm will output a best model with minimum number of variables (genes). The multivariate Cox regression analysis was also performed in R software and applied to further optimize and construct the final model. Finally, a prognostic signature based on five genes was established. The risk scores of UM samples were calculated by following formula:

$${\text{Risk}}\;{\text{ score }} = \, \beta {\text{1X1}} + \cdots + \beta {\text{iXi}}$$

where X represents the expression of a gene included in this prognostic model. β is the coefficient of a gene in the model.

After preparing the file containing the riskscores and survival time of patients, the survminer R package [23] could return an optimal cutoff value for best division of low- and high-risk groups who differ in their survival time. UM patients were then divided into a low- or a high-risk group by this optimal cutoff value of risk scores (1.7095) as the cutoff. To compare the survival rate between the low- and high-risk groups, we then performed log-rank test (Kaplan–Meier curve analysis) and log-rank *P* < 0.05 was taken as statistically significant. In addition, we used receiver operating characteristic (ROC) analysis to assess the predictive value of this signature. Furthermore, independence analysis of the signature with other clinical characteristics was conducted through univariate and multivariate Cox regression analyses and *P* < 0.05 was taken as statistically significant. Next, the predictive accuracy and sensitivity of this signature was evaluated in the testing cohorts including the GSE44295 and TCGA-UM datasets by ROC analysis.

### Establishment of a prognostic prediction nomogram

Nomogram is a convenient device for survival prediction of patients with cancers and now widely used in oncology research [24, 25]. In the current study, we constructed a nomogram to evaluate the 1-year, 2-year and 3-year OS probability of patients with UM in the training set.

### Gene Set Enrichment Analysis (GSEA)

The KEGG pathways enriched in the high-risk groups were identified by the GSEA method (https://pypi.org/project/gseapy/) and the gene lists involved in the pathways were also downloaded from the GSEA website [26].

### Tumor microenvironment (TME) analyses of low- and high-risk groups

ESTIMATE is a method for assessing the infiltration of stromal and immune cells in tumor tissues based on gene expression data [11]. We performed the ESTIMATE analysis by using the “estimate” R package, the gene expression matrix of UM patients and the reference files obtained from the developer of this algorithm, and finally got the stromal and immune score for each UM sample. CIBERSORT is an algorithm that can determine the relative proportions of 22 immune cell types within the leukocyte compartment in single tumor sample through using a set of 22 immune cell reference profiles (LM22) [12]. We assessed the infiltration levels of different immune cells in UM samples by using the “CIBERSORT” R program, the reference file obtained from the developer of this algorithm and the gene expression matrix of UM patients. 100 times was set for permutation test to ensure the accuracy of the results. The TIMER (Tumor IMmune Estimation Resource) is a tool for investigating different cancer and/or immune cells by using gene expression profiles [27]. We used the TIMER website (http://timer.cistrome.org/) to investigate the correlation between gene expression and infiltration of immune cells.

### Analysis of the somatic mutations in low- and high-risk groups

The visualization of somatic mutation landscape in low- and high-risk patients in TCGA-UVM cohort was performed and differentially mutated genes (DMGs) between these two groups were identified using maftools R package [28]. *P* < 0.05 was taken as statistically significant.

### Statistical analysis

All statistical *P* value were two-side and *P* < 0.05 was taken as statistically significant. Wilcoxon test was used to compare the differences between two groups. All analyses of data were conducted in R 4.0.1 software.

## Results

### Construction of the co-expression modules

**Fig. 1:**
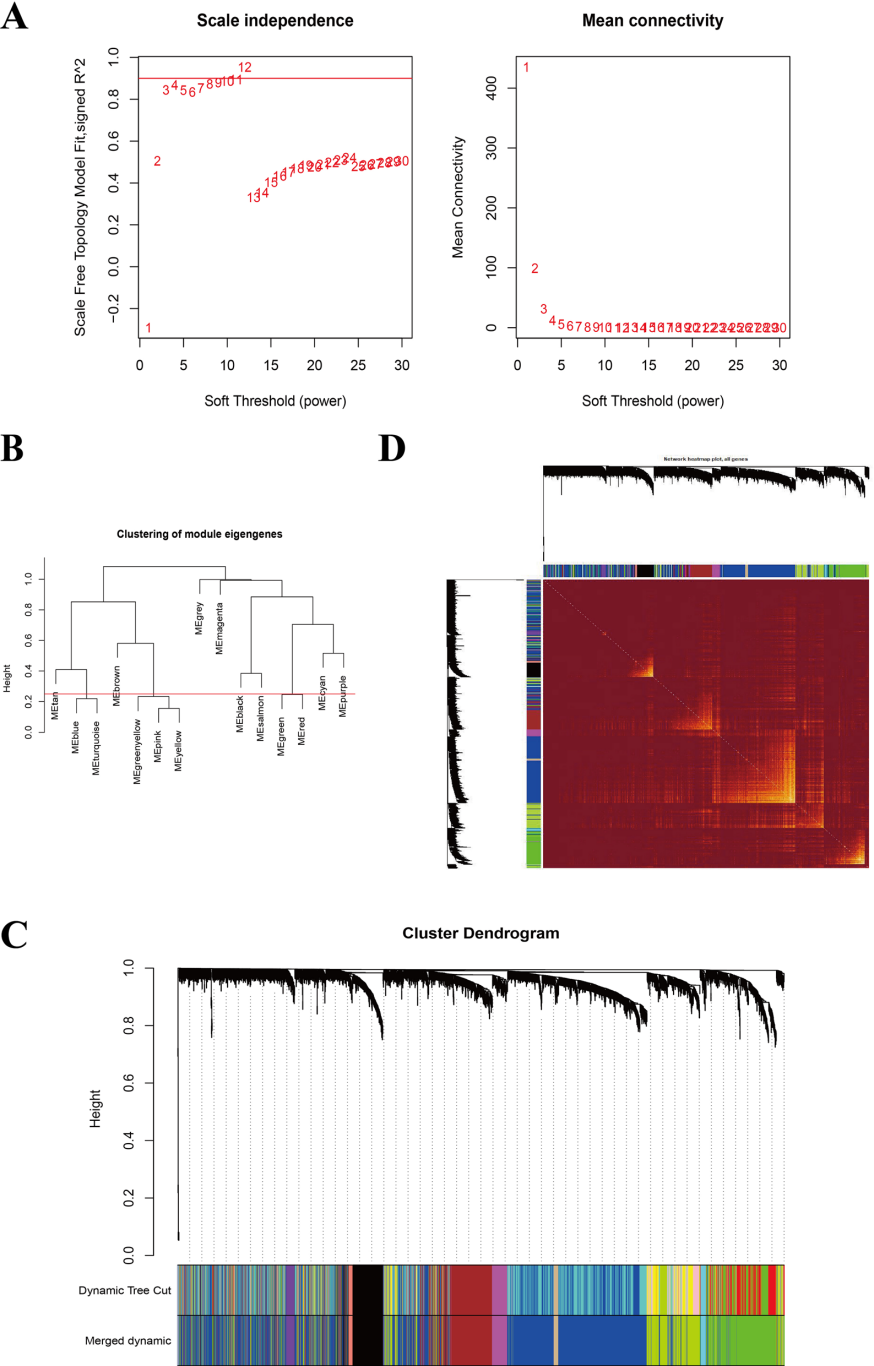
Construction of the co-expression modules via WGCNA in the training set. **A** Screening for power values. **B** 0.25 was chosen as the cut height threshold to merge possible similar modules. **C** Clustering dendrogram of co-expression modules. **D** Network heatmap plot of the modules

The basic clinical information of the patients in the three cohorts was presented in Table 1. The co-expression modules were constructed through the WGCNA method by using 3,322 genes generated from 63 UM samples in the training set GSE22138 (Fig. 1). Figure 1A–C showed the quality control process. Network heatmap plot of the modules was shown in Fig. 1D.

**Table 1:** The basic clinical information of 200 UM patients in three cohorts

Clinical traits	GSE22138 (n = 63)	GSE44295 (n = 57)	TCGA-UM (n = 80)
*Gender*			
Male	39 (61.9)	32 (56.1)	45 (56.25)
Female	24 (38.1)	25 (43.9)	35 (43.75)
*Age*			
< 65 years	36 (57.14)	–	–
≥ 65 years	27 (42.86)	–	–
*Metastasis*			
Yes	35 (55.56)	24 (42.1)	–
No	28 (44.44)	33 (57.9)	–
*TMN stages*			
II	–	–	39 (48.75)
III	–	–	36 (45)
IV	–	–	4 (5)
Unknown	–	–	1 (1.25)

### Correlation between co-expression modules and clinical characteristics

**Fig. 2:**
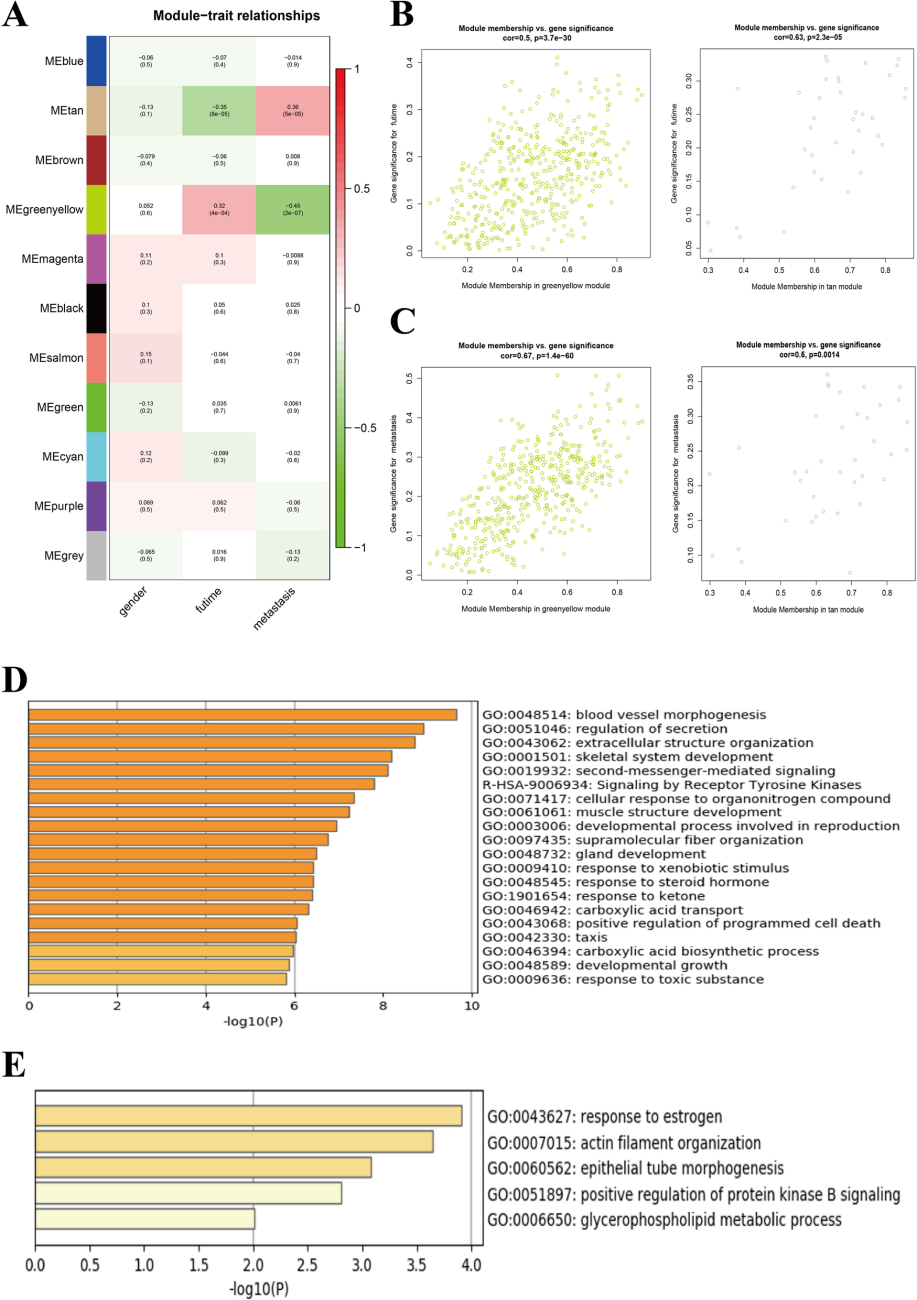
Co-expression modules mostly related to survival time and metastasis of UM. **A** Heatmap plot of the correlation between co-expression modules and clinic characteristics of UM. **B** The gene significance for survival time in the greenyellow module (left) and tan module (right). **C** The gene significance for metastasis in the greenyellow module (left) and tan module (right). **D** GO and KEGG enrichment analyses of genes in greenyellow module. **E** GO enrichment analysis of genes in tan module

The correlation between modules and clinical characteristics in the training set GSE22138 was presented in Fig. 2A. The greenyellow and tan modules were mostly related to futime (the full survival time of UM patients) and metastasis of UM patients (Fig. 2A–C). There were 421 genes in greenyellow module and 36 in tan module (Additional file 1: Table S1). The GO terms and KEGG pathways of genes in greenyellow and tan modules were shown in Fig. 2D, E, respectively. Genes in the greenyellow module were significantly enriched in the blood vessel morphogenesis, regulation of secretion, extracellular structure organization GO terms and signaling by receptor tyrosine kinases KEGG pathway. Genes in the tan module were gathered in the response to estrogen, actin filament organization, epithelial tube morphogenesis, positive regulation of protein kinase B signaling and glycerophospholipid metabolic process GO terms.

### Establishment of gene-based prognostic signature

**Fig. 3:**
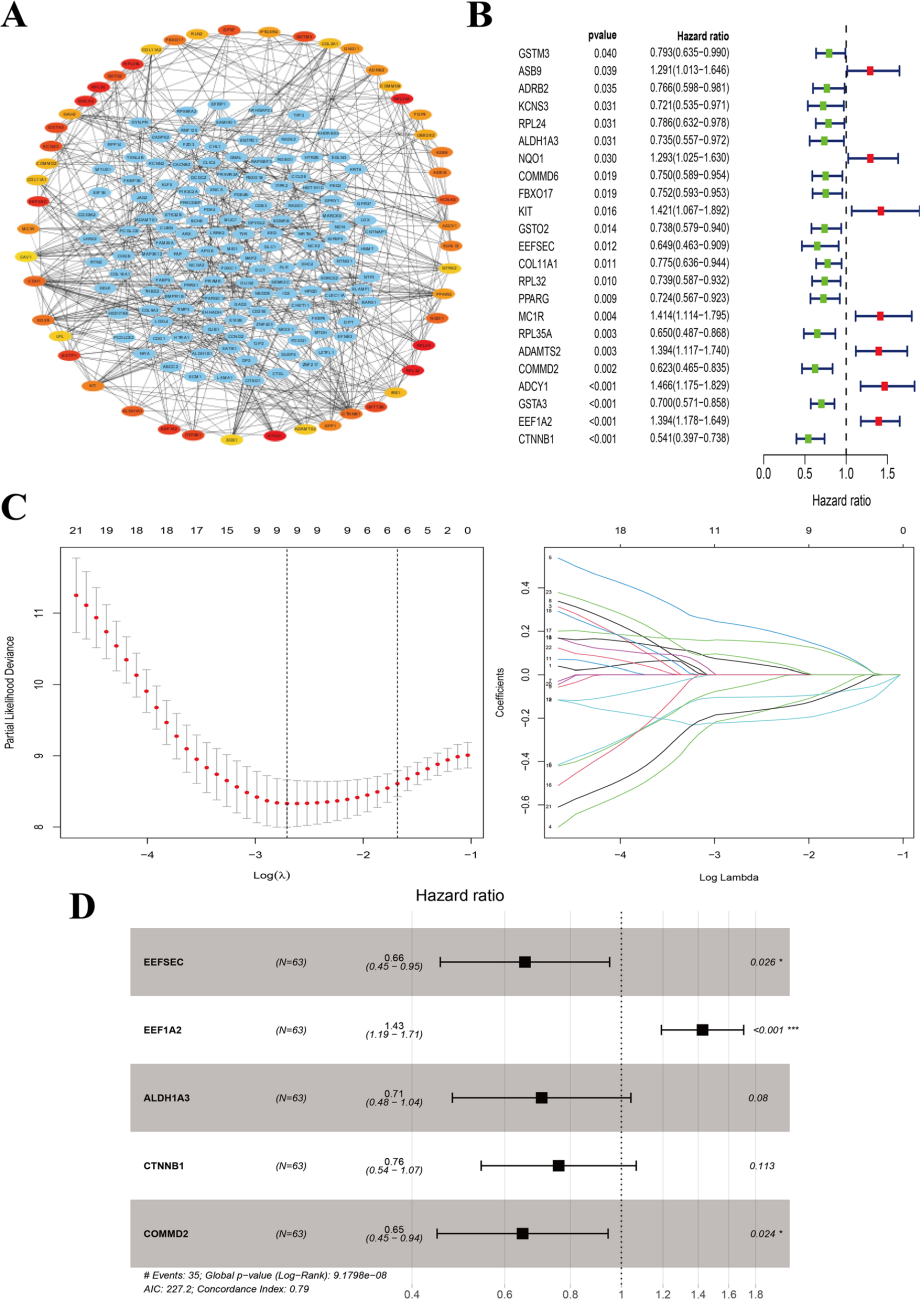
The establishment of gene-based prognostic signature for patients with UM in the training set. **A** The visualization of the top 50 hub genes in the greenyellow and tan modules. **B** OS-related genes among the hub genes identified by univariate Cox regression analysis (*P* < 0.05). **C** Cross-validation of parameter selection in Lasso-penalized Cox regression analysis. **D** The forest plot showed the Hazard ratio of enrolled survival-related genes

We further constructed a gene interaction network of the combination of the greenyellow and tan modules and identified top 50 hub genes in this network via MCC method in cytohubba app in Cytoscape software (Fig. 3A). Moreover, we identified genes related with overall survival (OS) of these 50 hub genes in the training set through univariate Cox regression test (*P* < 0.05) (Fig. 3B). Low expression of *GSTM3, ADRB2, KCNS3, RPL24, ALDH1A3, COMMD6, FBXO17, GSTO2, EEFSEC, COL11A1, RPL32, PPARG, RPL35A, COMMD2, GSTA3* and *CTNNB1* was correlated with poor survival, while for *ASB9, NQO1, KIT, MC1R, ADAMTS2, ADCY1 and EEF1A2*, high expression was correlated with poor survival in UM patients. We then aimed to use these 23 survival-related genes to construct a signature for OS predicting via Lasso-penalized and multivariate Cox regression analyses (Fig. 3C, D). Here, a prognostic signature consisting of 5 genes (*EEFSEC, EEF1A2, ALDH1A3, CTNNB1* and *COMMD2*) was established (Fig. 3D). Risk scores of UM patients were then calculated according to the formula mentioned above in the Material and Methods part. The genes and their coefficients were set in Table 2.

**Table 2:** The genes and their coefficients in the prognostic model

Gene symbol	Coefficient	HR	HR.95L	HR.95H
*EEFSEC*	− 0.422	0.656	0.453	0.950
*EEF1A2*	0.355	1.427	1.192	1.708
*ALDH1A3*	− 0.349	0.705	0.477	1.043
*CTNNB1*	− 0.274	0.760	0.541	1.067
*COMMD2*	− 0.432	0.649	0.446	0.944

### Assessment of prognostic value of the 5-gene signature

**Fig. 4:**
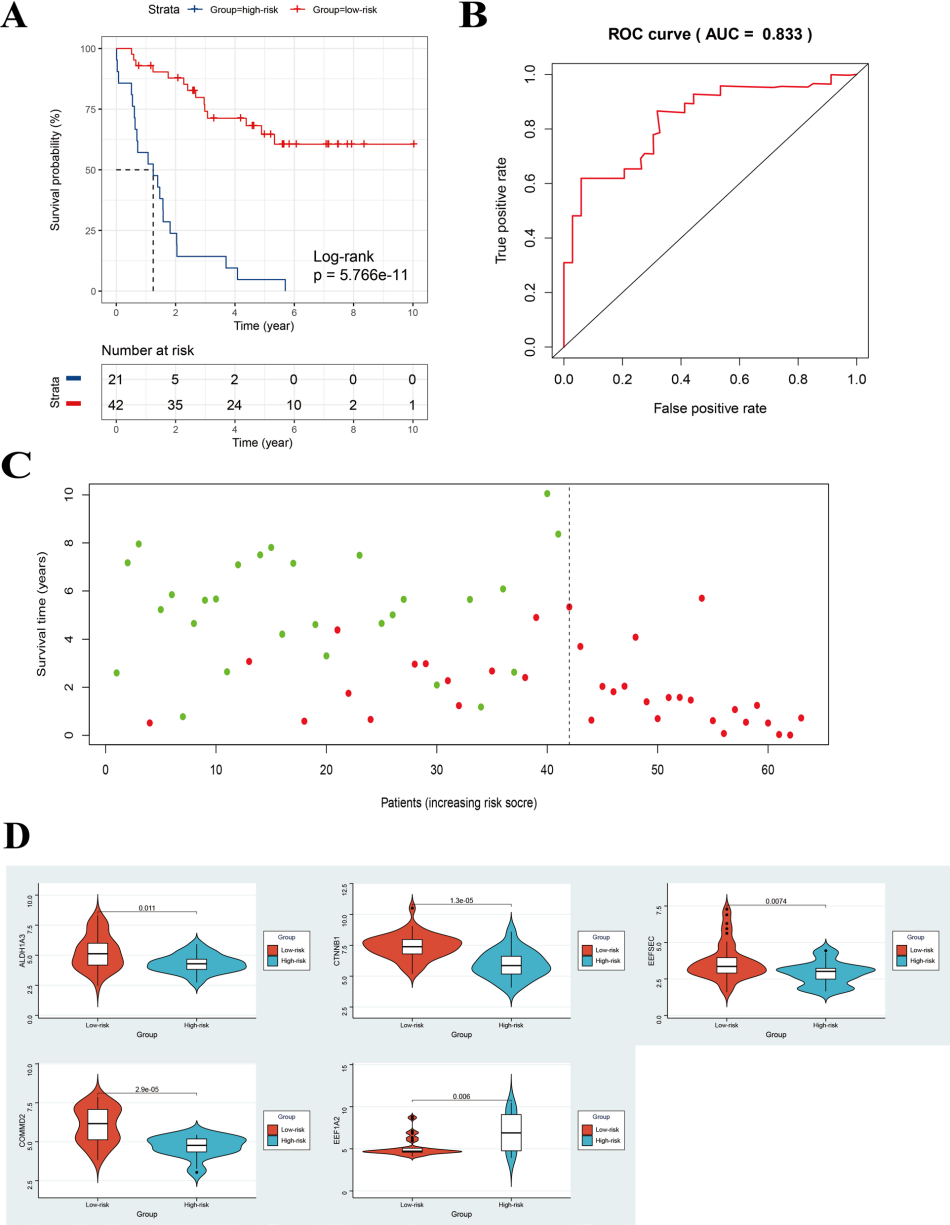
Assessment of prognostic value of the 5-gene signature in the training set. **A** Kaplan–Meier curve of patients with UM in the low- and high-risk groups of the training set GSE22138. **B** ROC curve for predicting accuracy of the 5-gene signature. **C** The risk scores and survival status of UM patients (one dot represents one UM patient, and the colors of the dots represent the survival status of patients; red color: dead, green color: alive). **D** The comparison of the expression of the five genes in low- and high-risk groups in the training cohort by Wilcoxon test. ROC, receiver operating characteristic

After construction of the 5-gene-based OS-predicting signature, UM patients in the training set GSE22138 were divided into low- and high-risk groups by using the risk scores calculated by the formula and the optimal cutoff value identified by survminer R package. The clinical characteristics of patients in training cohort in the two risk groups were presented in Table 3. The metastatic rate was higher in high-risk group (100%), which indicated that our gene signature could nicely predict the progression and aggressiveness of UM. Kaplan–Meier curve indicated that high-risk patients had significantly poorer OS than low-risk ones (log-rank *P* < 0.001) (Fig. 4A). We then performed univariate and multivariate Cox regression analysis to demonstrate the prognostic value of the riskscore calculated by the formula mentioned above. The results suggested that the riskscore was a prognostic factor for UM (Table 4).

**Table 3:** The clinical characteristics of patients in the two risk groups in GSE22138 cohort (n = 63)

Clinical characteristics	Low-risk group (n = 42)	High-risk group (n = 21)
*Gender*		
Male	27 (64.3)	12 (57.1)
Female	15 (35.7)	9 (42.9)
*Age*		
< 65 years	23 (54.8)	13 (61.9)
≥ 65 years	19 (45.2)	8 (38.1)
*Metastasis*		
Yes	14 (33.3)	21 (100)
No	28 (66.7)	0

**Table 4:** The results of univariate and multivariate Cox regression analysis for riskscore in GSE22138

Traits	HR	HR. 95L	HR. 95H	*P* value
*Univariate Cox regression analysis*				
Age	1.020803	0.994487	1.047816	0.122314
Gender	1.423855	0.71407	2.839167	0.315599
riskScore	1.170212	1.10997	1.233723	5.57E−09
*Multivariate Cox regression analysis*				
Age	1.00895	0.981786	1.036864	0.52226
Gender	2.108647	0.976914	4.551464	0.057372
riskScore	1.190268	1.119143	1.265913	3.01E−08

We performed ROC analysis to evaluate the prognostic value of the five-gene-based signature. The area under curve (AUC) of ROC was 0.833 (Fig. 4B). The survival status was plotted for each patient ranked by the risk scores which showed that the mortality of high-risk patients was much higher than low-risk ones (Fig. 4C). Moreover, we compared the expression of the five genes in the two risk groups and found that they all showed significant difference between these two risk groups (*P* < 0.05) (Fig. 4D). The expression of *EEF1A2* was higher and the expression of *EEFSEC, ALDH1A3, CTNNB1* and *COMMD2* was lower in high-risk group compared with low-risk group, which is consistent with the previous survival analysis of these five genes (Figs. 3B, 4D). We further compared the gene signature in the current study with other previously reported gene signatures (Table 5).

**Table 5:** The comparisons between our gene signature and other previously reported gene signatures

Function of genes	Number of genes	AUC of the signature	Origin
Metastasis-related	5	0.833	This study
Prognosis-related	18	0.803	Xue et al. [29]
Autophagy-related	9	0.907	Zhang et al. [30]
Immune-related	3	0.869	Gu et al. [31]
Ferroptosis-Associated	7	0.766	Luo et al. [32]
Immune-related	2	0.82	Li et al. [33]

### Validation of the 5-gene-based signature in the testing sets

We then tried to validate the prognostic value of our signature in external datasets. Firstly, risk scores of patients with UM in the testing sets including GSE44295 and TCGA-UM datasets were calculated by the formula derived from the 5-gene signature. The patients in the testing sets were subsequently divided into low- and high-risk groups by using the optimal cutoff value of risk scores obtained from the training set GSE22138. The clinical characteristics of patients in the two risk groups in the testing cohorts including GSE44295 and TCGA-UM were presented in Tables 6 and 7, separately. The metastatic rate was higher in high-risk group and the patients in high-risk group were found to be with higher TMN stage, which indicated that our gene signature could nicely predict the progression and aggressiveness of UM.

**Table 6:** The clinical characteristics of patients in the two risk groups in GSE44295 cohort (n = 57)

Clinical characteristics	Low-risk group (n = 35)	High-risk group (n = 22)
*Gender*		
Male	18 (51.4)	14 (63.6)
Female	17 (48.6)	8 (36.4)
*Metastasis*		
Yes	11 (31.4)	13 (59.1)
No	24 (68.6)	9 (40.9)

**Table 7:** The clinical characteristics of patients in the two risk groups in TCGA-UM cohort (n = 80)

Clinical characteristics	Low-risk group (n = 31)	High-risk group (n = 49)
*Gender*		
Male	18 (51.4)	27 (55.1)
Female	13 (48.6)	22 (44.9)
*T staging*		
T2	8 (25.8)	6 (12.3)
T3	14 (45.2)	18 (36.7)
T4	9 (29.0)	25 (51.0)
*M staging*		
M0	21 (67.7)	30 (61.2)
M1	-	4 (8.2)
Unknown	10 (32.3)	15 (30.6)
*N staging*		
N0	21 (67.7)	31 (63.3)
Unknown	10 (32.3)	18 (36.7)
*TMN stages*		
II	20 (64.5)	19 (38.8)
III	11 (35.5)	25 (51.0)
IV	-	4 (8.2)
Unknown	-	1 (2.0)

**Fig. 5:**
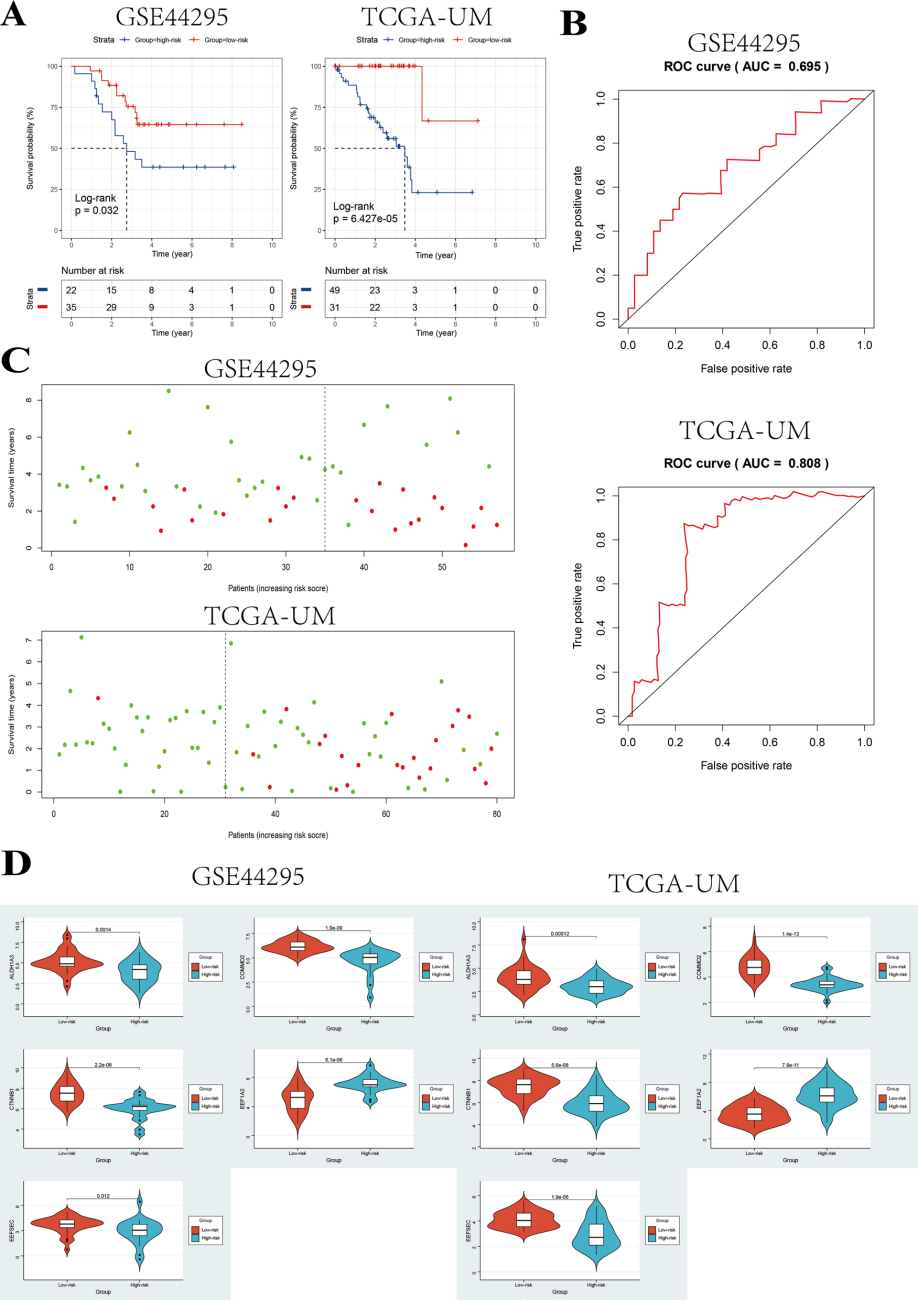
Validation of the 5-gene-based signature in the testing sets including GSE44295 and TCGA-UM datasets. **A** Kaplan–Meier curves of patients with UM in the low- and high-risk groups of the testing sets. **B** ROC curves for predicting accuracy of the 5-gene signature. **C** The risk scores and survival status of UM patients in the testing sets. **D** The comparison of the expression of the five genes in low- and high-risk groups in the two testing cohorts by Wilcoxon test

Kaplan–Meier curves indicated that high-risk patients had significantly poorer OS than low-risk ones in GSE44295 set (log-rank *P* < 0.05) and TCGA-UM set (log-rank *P* < 0.001) (Fig. 5A). Furthermore, we performed univariate Cox regression analysis and multivariate Cox regression and found that the riskscore was a prognostic factor for UM patients in GSE44295 and TCGA-UM cohorts (Tables 8, 9). The AUC of the 5-gene-based signature in GSE44295 and TCGA-UM cohorts was 0.695 and 0.808, respectively (Fig. 5B). The risk scores and survival status ranked by the risk scores of UM patients were then plotted for the testing sets (Fig. 5C). We further compared the expression of the five genes in the two risk groups and found that they all showed significant difference between these two risk groups (*P* < 0.05) (Fig. 5D). The expression of *EEF1A2* was higher and the expression of *EEFSEC, ALDH1A3, CTNNB1* and *COMMD2* was lower in high-risk group compared with low-risk group, which is consistent with the previous survival analysis of these five genes (Figs. 5D, 3B).

**Table 8:** The results of univariate and multivariate Cox regression analysis for riskscore in GSE44295 dataset

Traits	HR	HR. 95L	HR. 95H	*P* value
*Univariate Cox regression analysis*				
Age	0.998542561	0.968789443	1.029209447	0.924711066
Gender	1.711235909	0.73195866	4.000674487	0.215037537
riskScore	1.118781808	1.03738176	1.206569058	0.003589176
*Multivariate Cox regression analysis*				
Age	1.002774881	0.970596584	1.036019989	0.867747283
Gender	2.120960263	0.863152719	5.211676148	0.101185084
riskScore	1.143034477	1.05144219	1.242605469	0.0017064

**Table 9:** The results of univariate and multivariate Cox regression analysis for riskscore in TCGA-UM dataset

Traits	HR	HR. 95L	HR. 95H	*P* value
*Univariate Cox regression analysis*				
Gender	1.541871093	0.650966553	3.652056247	0.325023777
Stage	2.250353114	1.070583443	4.730214321	0.032362254
T stage	1.738860513	0.893333221	3.384667462	0.103518145
riskScore	1.040502389	1.017062328	1.064482669	0.000637195
*Multivariate Cox regression analysis*				
Gender	1.436613038	0.5955889	3.465237552	0.419984413
Stage	1.654894219	0.64058205	4.275291316	0.298228898
T stage	1.129647256	0.492554011	2.590787804	0.773462992
riskScore	1.036907481	1.01310206	1.061272273	0.002224966

### Establishment of the nomogram

**Fig. 6:**
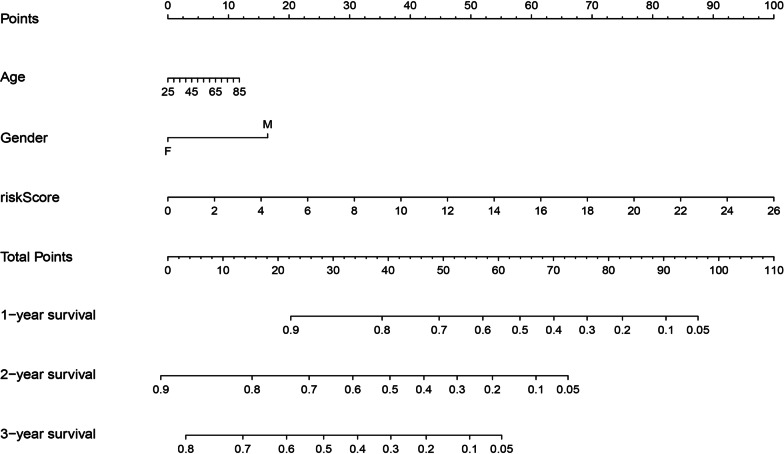
The nomogram for better prognosis prediction for UM patients based on the Cox regression analyses results from the training set. The total number of scores project on the bottom scale represents the 1, 2 and 3-year OS probability. OS, overall survival

To meet clinical needs, we constructed a nomogram by using the multivariate Cox regression analysis results mentioned above in the training set (Fig. 6).

### GSEA of high-risk groups

**Fig. 7:**
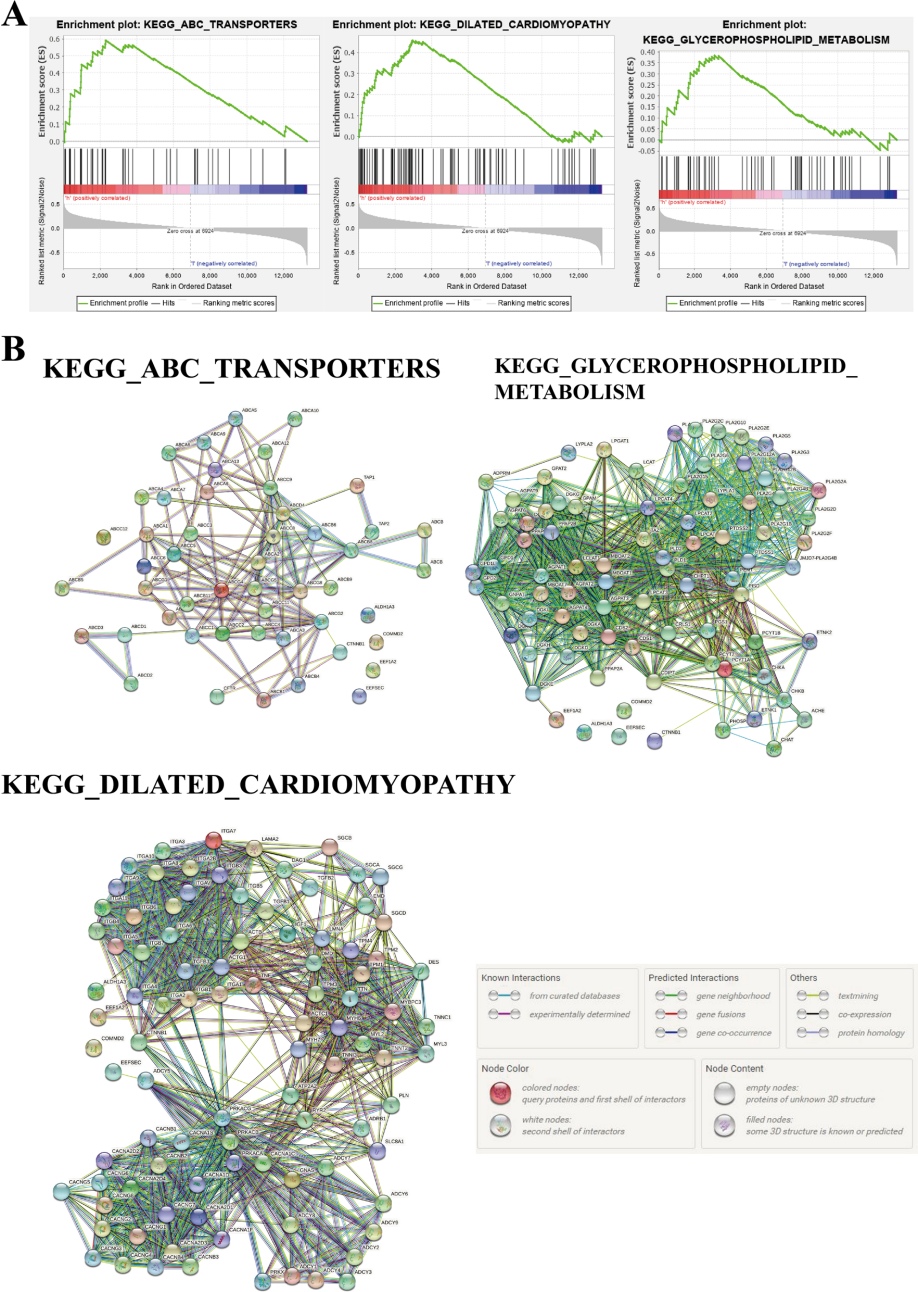
GSEA analysis of the high-risk groups. **A** Top 3 KEGG [15–17] pathways ranked by the NES values enriched in the high-risk group. **B** The interaction networks of the five genes in the gene signature and genes in the three identified pathways, obtained from String database

After the construction of the gene signature, UM patients could be divided into low- or high-risk group. To identify important pathways involved in the development of UM, we performed GSEA analysis and found that the top 3 KEGG pathways enriched in the high-risk group were ABC_TRANSPORTERS, DILATED_CARDIOMYOPATHY and GLYCEROPHOSPHOLIPID_METABOLISM with nominal *P* value  < 0.05 (Fig. 7A). We then downloaded the gene lists of these three pathways from the GSEA website and evaluated the interactions between the five genes in the gene signature and genes in the three identified KEGG pathways. The results from the String database showed that *CTNNB1* is interacted with genes in all three KEGG pathways (Fig. 7B), which indicated that this gene might play an important role in the high-risk behavior of UM.

### Tumor microenvironment (TME) analyses of the low- and high-risk groups

**Fig. 8:**
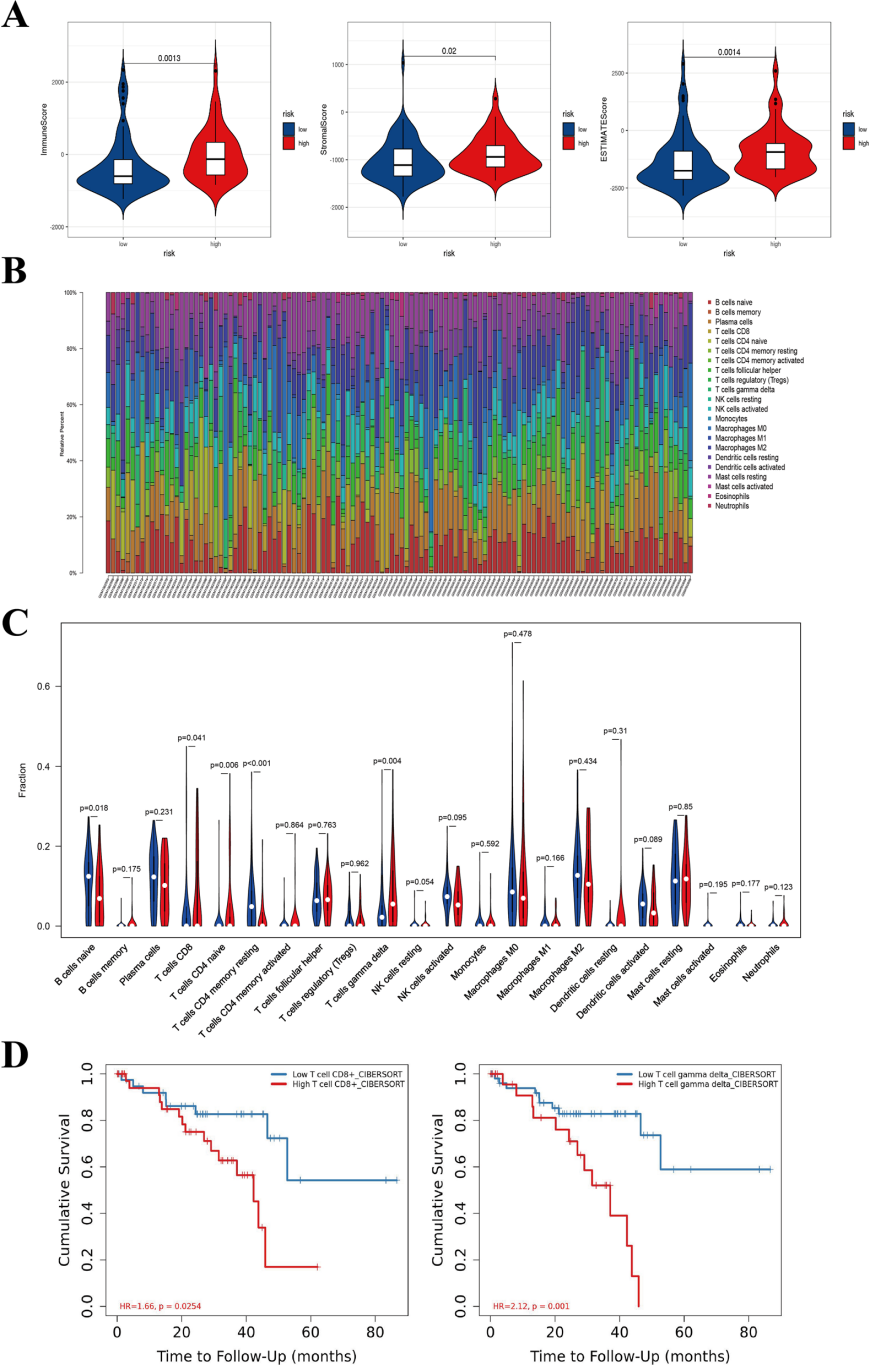
TME analyses of the 120 UM samples in the GSE22138 and GSE44295 datasets and comparison between the low- and high-risk UM samples. **A** The immunescore and stromalscore of high-risk UM samples were significantly higher than that of the low-risk ones (*P* < 0.05). **B** The visualization of infiltration levels of immune cells in the TME of 120 UM samples. **C** The comparison of infiltration levels of different immune cells between the low- (blue bar) and high-risk (red bar) UM samples. **D** The correlation between infiltrating immune cells and OS in UM identified by using the TIMER website. T cells CD8 and T cells gamma delta are significantly associated with OS of UM patients (*P* < 0.05). TME, tumor microenvironment. TIMER, Tumor IMmune Estimation Resource

The TME of 120 UM samples in the GSE22138 and GSE44295 datasets were assessed via ESTIMATE algorithm. The immune-score and stromal-score of the high-risk UM samples were significantly higher than that of the low-risk ones (Fig. 8A). We then used the CIBERSORT algorithm to determine the infiltration levels of immune cells in the TME of UM samples (Fig. 8B) and compared the results of the high-risk samples and low-risk ones. The results indicated that the infiltrating levels of T cells CD8 and T cells gamma delta were significantly higher in high-risk UM group compare with low-risk group (*P* < 0.05) (Fig. 8C). Furthermore, high infiltration of T cells CD8 and T cells gamma delta were found to be associated with worse OS of UM patients (*P* < 0.05) by using the TIMER website (Fig. 8D). High-risk UMs were with worse prognosis and higher infiltrating levels of T cells CD8 and T cells gamma delta, indicating that the bad prognosis was might be partly caused by the high infiltration of these two types of immune cells, which was consistent with previous studies [34, 35]. Notably, *EEF1A2* was slightly positively correlated with T cells gamma delta and T cells CD8, whereas *CTNNB1* was slightly negatively correlated with T cells CD8 and T cells gamma delta (*P* < 0.05) (Additional file 2: Figure S1). *EEFSEC* only showed a slightly negative correlation with T cells gamma delta (Additional file 2: Figure S1B).

### The somatic mutation landscape in low- and high-risk groups

**Fig. 9:**
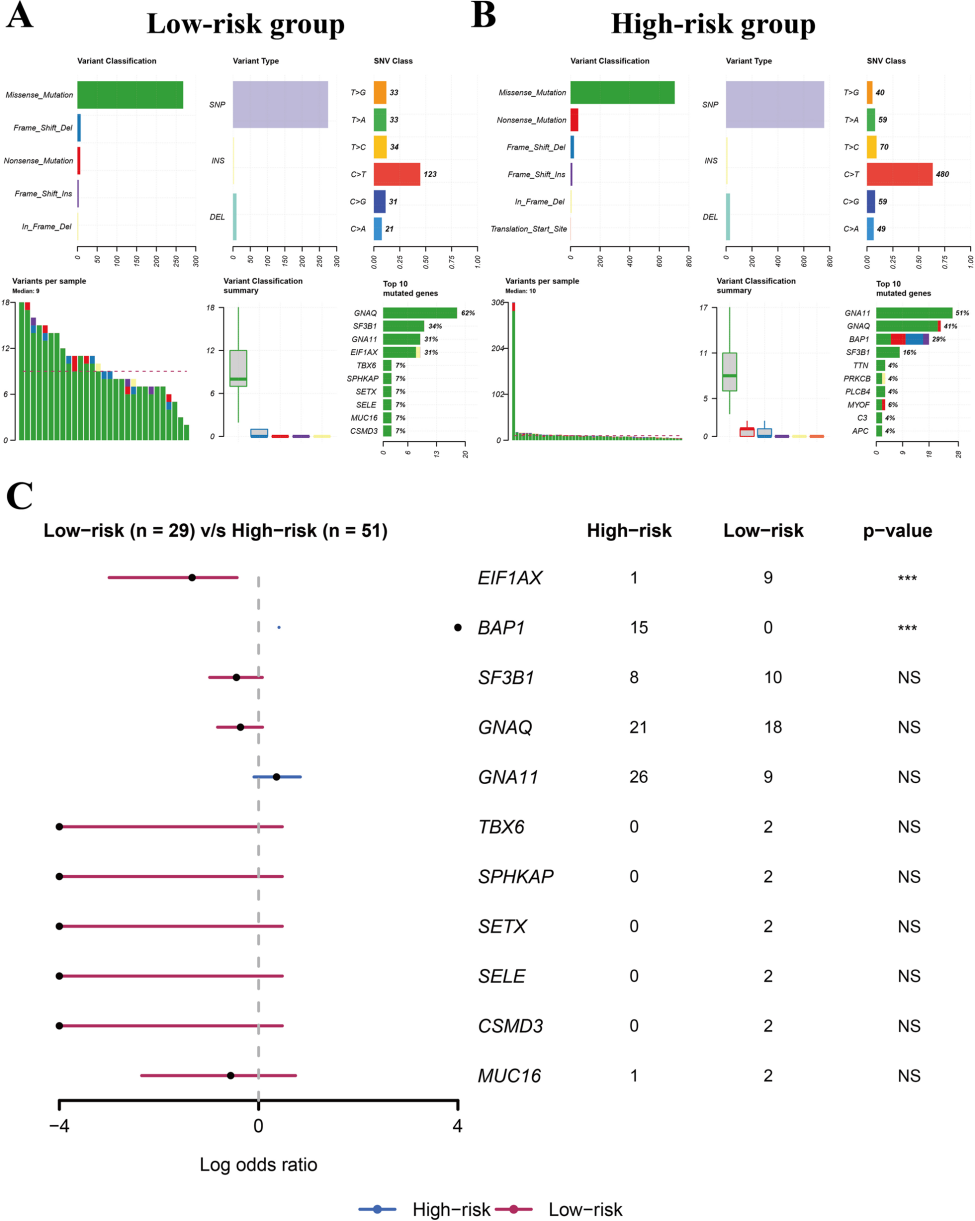
The analysis of somatic mutations in low- and high-risk groups. **A** The summary of somatic mutation landscape in low-risk group. **B** The summary of somatic mutation landscape in high-risk group. **C** The differentially mutated genes between low- and high-risk groups

We then evaluated the differences in the somatic mutation landscape between low- and high-risk groups in TCGA-UVM cohort. These two groups showed different somatic mutation landscapes (Fig. 9) and the high-risk group was found to be with higher mutation frequency of BAP1 (Fig. 9c).

## Discussion

UM is the most common intraocular malignant tumor in adults where almost half of the patients with UM develop systemic metastasis despite successful local control. The systemic metastasis of UM commonly involves the liver. Patients with metastatic disease have a poor prognosis, which is generally fatal within 1 year after confirming as metastases. Prognosis prediction of UM patients affects the choices of further treatment options. Therefore, identifying the underlying mechanisms of its metastasis and biomarkers with prognostic value would help clinicians and patients to make better treatment decisions. Michael D. Onken et al. assessed the prognostic prediction value of the gene-expression data for UM patients by through a series of studies [36, 37]. Their results indicated that gene-based OS-predicting signature was a promising and reliable tool for UM patients and clinicians.

Several studies have focused on prognosis prediction based on the gene expression profiles of UM. A prognostic 15-gene expression profile (15-GEP) test has been in clinical use for several years to predict metastatic risk [37, 38]. Aaberg TM et al. [39, 40] reported that the 15-GEP helps predict the metastatic risk of UM and guides the management of these patients. Binkley EM et al. investigated the effect of tumor size and thickness on the prognosis of UM patients [41]. Their study revealed that tumor size combined with the 15-GEP could predict the prognosis of UM patients. Afshar AR et al. [42] used the UCSF500 assay to detect genetic alterations including gene mutations and chromosomal copy number changes in UM and investigated the correlation between UCSF500 results and metastasis. Their results indicated that the chromosomal copy number changes and gene mutations detected by UCSF500 were strongly correlated with metastasis predictors, including the 15-GEP. Many other studies investigated the utility of the 15-GEP alone or in combination with other clinical or pathological factors in predicting the metastatic risk and prognosis [43–51]. Collectively, these studies have suggested that this 15-GEP could predict the prognosis and metastatic risk of UM patients. However, we found that the accuracy and potency of the 15-GEP alone in predicting prognosis were not high and can be improved further by combining it with other clinical or pathological characteristics. We identified metastasis-related genes and focused on constructing a gene signature based on these genes for better prognosis prediction. It is difficult to claim superiority for our gene signature relative to the 15-GEP. However, our results might provide an alternative to serving the clinical practice.

In the past five years, several studies tried to identify essential genes involved in the development of UM [29, 31, 32, 34, 35, 52–54]. These studies identified different aspects of UM such as clinical and pathological status along with the TME. However, they vary in the selection of bioinformatic tools and essential genes. These studies used bioinformatics tools including WGCNA, immune analysis methods such as ESTIMATE and CIBERSORT, and unsupervised clustering. As these studies utilized different tools to identify essential genes and enrolled different genes into the gene signatures, predictable and obvious differences exist in their outcomes. Moreover, these study results might be complementary to each other. Metastasis, a very important event in the development of uveal melanoma, significantly affects the survival of patients. Of note, to date, no studies have used WGCNA, which is a widely used and helpful tool in cancer research, to identify metastasis-related genes and use these genes to construct a gene signature in uveal melanoma.

In the current study, eleven modules consisting of more than 20 co-expression genes were constructed from 63 UM samples in the GSE22138 dataset. The tan and greenyellow modules were significantly associated with the survival time and metastasis in UM. Among these identified metastasis-related genes, *BAP1* and *PRAME* were found in the greenyellow module. Moreover, *BAP1* and *PRAME* are strongly implicated in the progression and aggressiveness of UM [55–57], which strengthens their role in the advancement of UM. Notably, the mutations in *BAP1* are associated with the higher metastatic rate of UM [58]. Additionally, the results in this study showed that the mutation frequency of *BAP1* was higher in the high-risk group (Fig. 9C), which partly explains the worse prognosis of patients in this group. *SF3B1* has been shown to be associated with prognosis of UM patients [59] and the mutations in *SF3B1* are related to late onset of metastasis in UM [60]. However, *SF3B1* was not identified as a metastasis-related gene in this study, and the mutation frequency was not different between the low- and high-risk groups. Nonetheless, despite these findings, the contribution of *SF3B1* in the metastasis of UM cannot be overlooked. Moreover, the results might have been due to the limitation of the algorithms used in this study. *EIF1AX* has also been discussed in UM and mutations in this gene were associated with low-inflammation phenotype and low metastatic potential [1]. Of note, the mutation frequency of *EIF1AX* was lower in high-risk group identified by our gene signature. The results in our study showed that the high-risk group had a high-immune-infiltration phenotype and a poor prognosis with a higher metastatic rate. Therefore, the results in the current study were interestingly consistent with previous studies and highlighted the involvement of *EIF1AX* in UM.

Furthermore, we identified the top 50 hub genes of these two modules by Cytoscape software and enrolled them into the subsequent Cox regression analyses. The univariate Cox regression test result revealed that the *GSTM3, ASB9, ADRB2, KCNS3, RPL24, ALDH1A3, NQO1, COMMD6, FBXO17, KIT, GSTO2, EEFSEC, COL11A1, RPL32, PPARG, MC1R, RPL35A, ADAMTS2, COMMD2, ADCY1, GSTA3, EEF1A2* and *CTNNB1* were significantly associated with the OS of UM patients. We then constructed an OS-predicting signature via Lasso-penalized and multivariate Cox regression analysis by using expression profiles of these 23 genes. Finally, a prognostic signature consisting of 5 genes (*EEFSEC, EEF1A2, ALDH1A3, CTNNB1* and *COMMD2*) was established. The Cox regression analyses results revealed that *EEF1A2* was a high-risk factor in UM, while the overexpression of *EEFSEC, ALDH1A3, CTNNB1, COMMD2* were associated with a longer OS. In addition, Cox regression analyses results indicated that the 5-gene signature was an independent prognostic factor for UM patients. Notably, the survival curves and ROC analysis results of the training and testing sets showed the robustness and reliability of the signature for prognosis prediction of UM patients. The AUC of the ROC curves in the three cohorts were 0.833, 0.695 and 0.808, respectively. Other studies have reported variable gene signatures for predicting the OS of UM patients. The AUC of the ROC of the gene signature constructed by Xue et al. was 0.8 [29], Zheng et al. was 0.9 [30], Gu et al. was 0.869 [31], Luo et al. were 0.766 and 0.732 [32], and Li et al. was 0.82 [33]. Although the AUC value of the ROC curves identified in our study was not the highest among them, was not even the lowest, which indicated that our gene signature was potentially significant. Moreover, patients in the high-risk group identified by our gene signature had a higher metastatic rate (Tables 3, 6, 7). This finding suggested that our gene signature might identify UM patients with high metastatic risk. Furthermore, a nomogram was established to predict the 1, 2 and 3-year OS probability of UM patients.

*EEFSEC* has been identified as a modulator of arsenic trioxide (AsIII) toxicity in the treatment of chronic myeloid leukemia (CML) [61]. However, the role of *EEFSEC* in tumorigenesis and progression has not been well investigated. In our study, based on the univariate Cox regression analysis, *EEFSEC* was considered as a protective factor for the prognosis of UM. The overexpression of *EEF1A2* was found in prostate cancer and could be used as a biomarker for its risk-stratification [62]. *EEF1A2* was also identified as a biomarker with a significant prognostic value for UM in our study. Dysregulation and mutations of *CTNNB1* participate in the occurrence and progression of multiple tumors [63, 64]. Moreover, in the current study, these two genes were related to a better prognosis of UM patients. High expression of *ALDH1A3* has been associated with the worse OS in various tumors, including glioma and pancreatic cancer [65, 66]. In contrast, for metastatic *BRAF*-mutant melanoma, *ALDH1A3* expression was correlated with the better OS [67]. Interestingly, in the present study, the Cox regression analysis revealed that *ALDH1A3* was related to a better prognosis of UM patients (Hazard ratio = 0.735, *P* < 0.05).

After the risk scores calculation of UM samples, they were divided into the high- and low-risk groups by using the optimal cutoff value of risk scores identified by the survminer R package. Finally, we evaluated the TME of UM samples by using ESTIMATE and CIBERSORT algorithms to investigate further the factors influencing the prognosis of patients with UM. We found that the stromal and immune-scores of high-risk patients in the GSE22138 and GSE44295 datasets were significantly higher than those of low-risk patients. Moreover, T cells CD8 and T cells gamma delta infiltration levels were significantly higher in high-risk UM samples than low-risk ones (*P* < 0.05). Consistent with previous reports [34, 35], T cells gamma delta and T cells CD8 were identified to be related to poor prognosis in UM patients. The results indicated that the poor prognosis of high-risk patients might be partly caused by the high infiltration levels of T cells gamma delta and T cells CD8. Furthermore, the correlation between these two immune cells and the 5 genes in our prognostic signature were studied to prove the importance of these 5 genes. Notably, *EEF1A2* was slightly positively correlated with T cells gamma delta and T cells CD8, whereas *CTNNB1* was slightly negatively correlated with the T cells gamma delta and T cells CD8 (*P* < 0.05). On the other hand, *EEFSEC* only slightly negatively correlated with T cells gamma delta. These results suggested that the overexpression of *EEF1A2* might be partially responsible for the elevated infiltration levels of T cells gamma delta and T cells CD8, which makes it a high-risk factor in UM. While for *EEFSEC* and *CTNNB1*, the overexpression was slightly related to the decreased infiltration levels of T cells CD8 and T cells gamma delta. The data suggested their protective role in the prognosis of UM patients.

In conclusion, through WGCNA, we identified two co-expression modules including the tan and greenyellow modules significantly related to the survival time and metastasis of UM patients. Moreover, our five-gene-based prognostic signature is a stable and reliable tool for the OS-prediction of UM patients. The *EEF1A2, EEFSEC* and *CTNNB1* may influence the prognosis of UM patients through their effect on the infiltration levels of T cells gamma delta and T cells CD8.

## Conclusions

Our integrative analysis identified 23 key genes, which were significantly related to the metastasis and the prognosis of UM. Additionally, a prognostic signature was established based on the expression of these genes. These 23 genes might be important targets for investigating the mechanism underlying the metastasis of UM and the prevention of UM metastasis. Furthermore, our five-gene-based prognostic signature is a stable and reliable tool for OS-prediction of UM patients. The *EEF1A2, EEFSEC* and *CTNNB1* in our gene signature might influence the prognosis of UM patients through their influence on the infiltration levels of T cells gamma delta and T cells CD8.

## Supplementary Information

**Additional file 1. Table S1.** Metastasis-related genes identified by WGCNA.

**Additional file 2. Figure S1.** The relationship between the genes in our signature and survival-related immune cells.

## Data Availability

The datasets analyzed during the current study are available in the GEO database (https://www.ncbi.nlm.nih.gov/geo/) (PERSISTENT ACCESSION NUMBER TO DATASETS: GSE22138, GSE44295) and TCGA database (https://portal.gdc.cancer.gov/) (PERSISTENT ACCESSION NUMBER TO DATASET: TCGA-UVM). All data generated or analyzed during this study are included in this published article and its supplementary information files.

## Abbreviations

UM: Uveal melanoma
TME: Tumor microenvironment
TCGA: The Cancer Genome Atlas
GEO: Gene Expression Omnibus
CIBERSORT: Cell-type Identification By Estimating Relative Subsets Of RNA Transcripts
ESTIMATE: Estimation of STromal and Immune cells in MAlignant Tumor tissues using Expression data
KEGG: Kyoto Encyclopedia of Genes and Genomes
GO: Gene Ontology
OS: Overall survival
GSEA: Gene Set Enrichment Analysis
ROC: Receiver operating characteristic
