# Supplementary material for: Integrative analysis identifies key genes related to metastasis and a robust gene-based prognostic signature in uveal melanoma
Source: BMC Med Genomics. 2022 Mar 17;15:61. doi: 10.1186/s12920-022-01211-1 (PMC8932077; doi:10.1186/s12920-022-01211-1)
Supplement: Supplementary file 1 — Additional file 1. Table S1. Metastasis-related genes identified by WGCNA. [file 12920_2022_1211_MOESM1_ESM.docx]

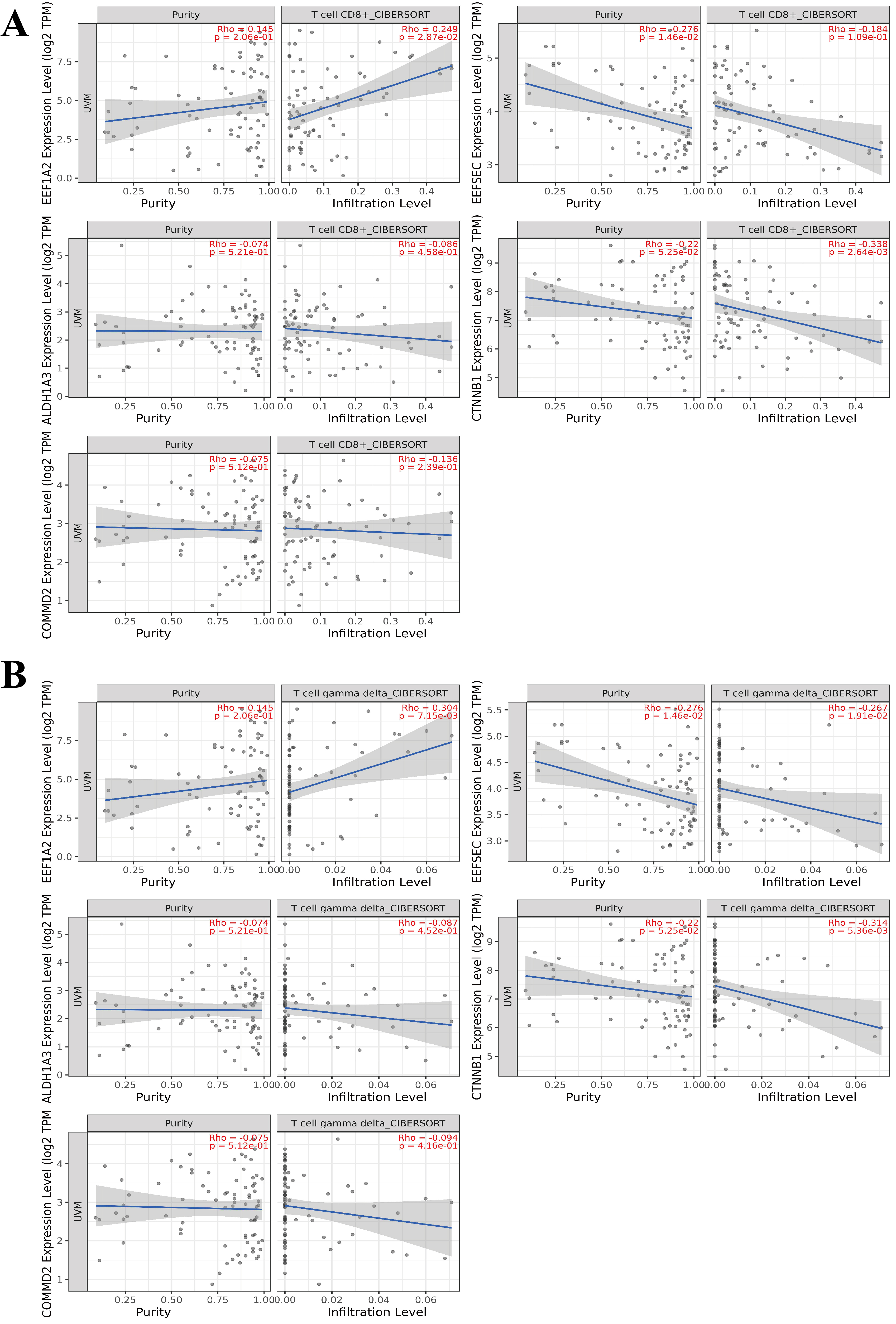


Figure S1. The relationship between the five genes in our signature and survival-related immune cells identified by using the TIMER website. (A) EEF1A2 was slightly positively correlated with T cells CD8, while CTNNB1 was slightly negatively correlated with T cells CD8 (*P* < 0.05). (B) EEF1A2 was slightly positively correlated with T cells gamma delta, whereas CTNNB1 and EEFSEC were slightly negatively correlated with T cells gamma delta (*P* < 0.05).
